# Invasive cellular blue nevus in the cervical spine: A case report

**DOI:** 10.1097/MD.0000000000037097

**Published:** 2024-02-09

**Authors:** Xianfeng Rao, Zhengwen Kang, Jianwei Chen, Tong Wang, Mengyao Ma, Shuwen Yang, Zetao Wu, Bo Wang, Qiusheng Zhang

**Affiliations:** a Department of Neurosurgery, Shenzhen Second People’s Hospital, Shenzhen University First Affiliated Hospital, Clinical College of Shantou University Medical College, Shenzhen, China; b Department of Pathology, Shenzhen Second People’s Hospital, Shenzhen University First Affiliated Hospital, Shenzhen, China.

**Keywords:** blue nevus, extramedullary, meningeal melanocytoma, meningeal melanoma, tumor

## Abstract

**Introduction:**

Cellular blue nevus is an uncommon neoplasm in the spine.

**Patient concerns:**

Here, we present a case of a 24 years old male with a 2 months history of numbness in the right upper limb and shoulder.

**Diagnosis:**

Cervical spine and subcutaneous tissue invasive cellular blue nevus.

**Interventions:**

The patient underwent C4 laminectomy and partial C3 and C5 laminectomy for total resection of the lesion. Histopathology revealed a nodular tumor with unclear boundaries, which was composed of heavily pigmented dendritic cells and more pigmented spindle cells.

**Outcomes:**

There was no recurrence during 3 years follow-up.

**Conclusion:**

Invasive cellular blue nevus of the spine can be wrongly diagnosed as spinal meningeal melanocytoma and meningeal melanoma due to its special cell behavior and rarity. Therefore, it is important to understand its pathological and clinical characteristics to avoid over-treatment.

## 1. Introduction

Blue nevus represents a broad spectrum of melanocytic proliferation with distinctive clinical and histopathological features, which classified several subtypes, including, common blue nevus and cellular blue nevus. Common blue nevus was first described by Jadassohn-Tieche and nevus often occur in the skin, oral mucosa, vagina, prostate, and conjunctiva.^[1]^ Histopathologically, common blue nevus are characterized by wedge-shaped, variable cellular dermal proliferation of distinctive spindled, dendritic melanocytes with elongated, hyperchromatic, nuclei and variable amounts of coarse intracytoplasmic pigment.^[2]^ Cellular blue nevus is a type of blue nevus that is characterized histologically by a cellular appearance and presents itself with subcutaneous infiltration, intensive pigmentation, and a large size.^[3]^ Pigmented tumors are not common in the central nervous system. Moreover, invasive cellular blue nevus has invasive growth model, and it is difficult to differentiate between blue nevus lesions and melanoma in clinical and histological aspects.^[4]^ In this report, a case of a patient with invasive cellular blue nevus in the cervical spine and subcutaneous is presented.

## 2. Case presentation

### 2.1. Clinical history

A 24-year-old Chinese male was admitted with a 2 months history of numbness in the right upper limb and shoulder. The remaining extremities were negative of any symptoms. No significant personal medical history and family history of systemic pigmented disease was reported. Examination identified decreased superficial sensation of the right upper limb and shoulder. No pigmented nodule or nevus was found in any skin or mucous. The functions of cranial nerves and the motor and sensory functions of the other extremities were intact.

### 2.2. Radiological examinations

**Figure 1.**
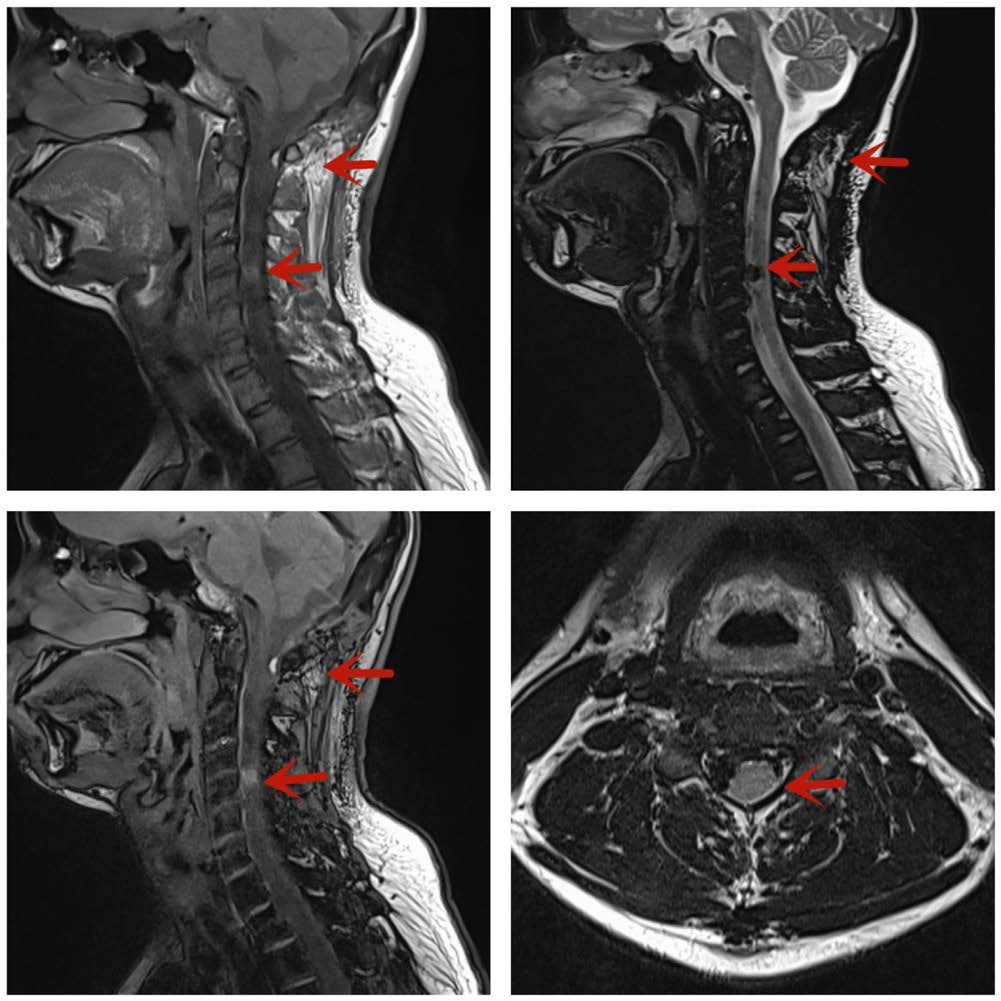
Magnetic resonance imaging (MRI) of the cervical spine showed a 10 × 7 mm cystic extramedullary mass between the C4 and C5 level, which was hyperintensity on T1-weighted imaging (T1WI), hypo-isointensity on T2WI and homogeneously enhanced with gadolinium diethylpentoic acid.

Magnetic resonance imaging of the cervical spine showed a 10 × 7 mm cystic extramedullary mass between the C4 and C5 level, which was hyperintensity on T1-weighted imaging and hypo-isointensity on T2WI. Moreover, another 6 × 3 mm subcutaneous mass at the C1 level was identified with the similar MR intensity on T1 and T2 imaging as the former mass. Both were homogeneously enhanced with gadolinium diethylpentoic acid (Fig. 1). Magnetic resonance imaging of the brain and thoracic spine revealed no abnormalities. The vertebrate and spine inC2–C5 level was normal.

### 2.3. Surgical findings and postoperative course

**Figure 2.**
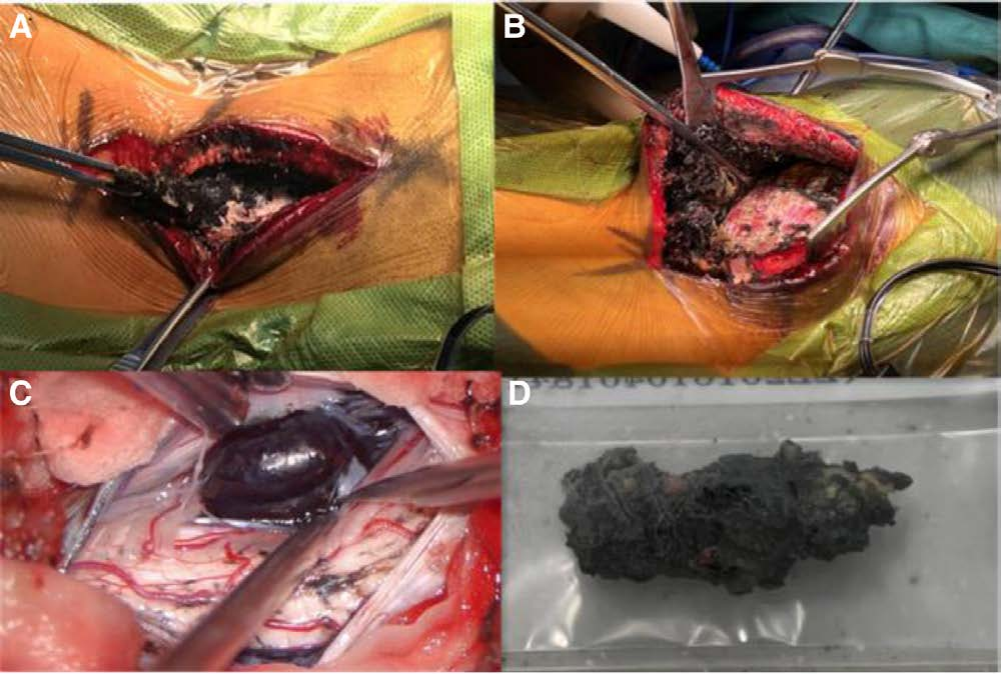
Intraoperative image.Subcutaneous mass at C1 (A) and C2-C5 (B).Subdural mass (C).Totally removed mass (D).

The patient underwent C4 laminectomy and partial C3 and C5 laminectomy for total resection of the lesion. Intraoperatively, the skin over the lesion was normal, a black lesion with clear mass and unclear boundaries was identified at the C1 level (Fig. 2A); the subcutaneous muscles and adipose tissue between C2-C5 also exhibited the same properties (Fig. 2B); intraoperative photograph showed pigmentation in the duration. A black tumor was seen on the flank of the cord at C4 level; the tumor was elastic and hard, adhered to the spinal cord, had limited blood supply (Fig. 2C), and was totally removed through midline durectomy (Fig. 2D);there was a close relationship between subcutaneous and epidural masses. Postoperatively, the numbness of the patient recovered. Presently, 4 years after the operations, and the patient ambulates freely, cervical spine magnetic resonance imaging reveals no relapse.

### 2.4. Pathological examination results

**Figure 3.**
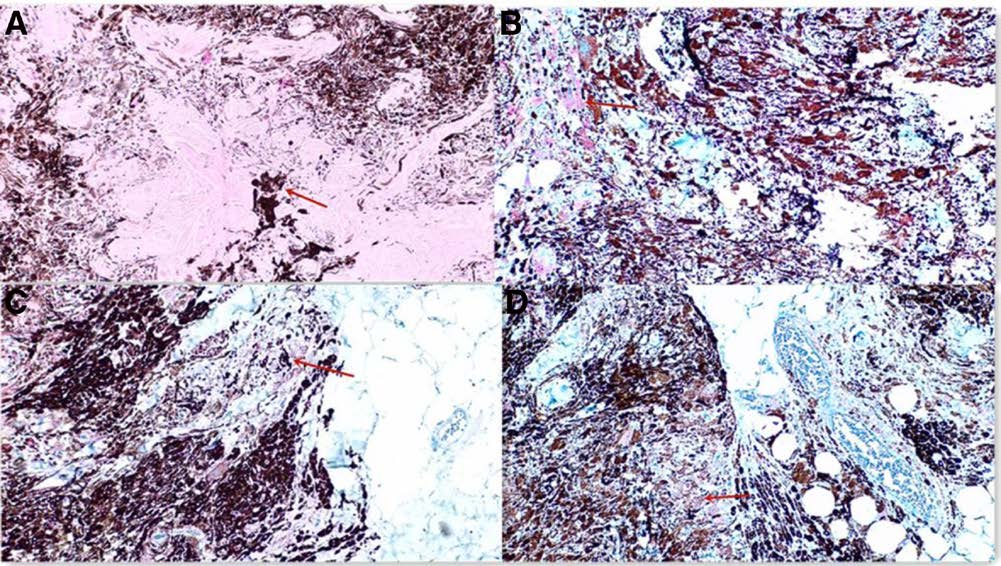
(A) Tumor tissues were an uneven distributed pigment and without invading of the surrounding epidermis,) the dendritic cells with abundant pigmented, the spindle cells with slightly pigmented(hematoxylin-eosin; magnification × 100). (B) Immunohistochemical staining of blue nevus cells with melan-A shows stained (magnification × 100). (C) Immunohistochemical staining of blue nevus cells with HMB45 shows strongly stained.(magnification × 100). (D) Immunohistochemical staining of blue nevus cells with Ki-67 shows lowly stained (magnification × 100).

Histopathological examination of the pigmented lesion in the subcutaneous specimens revealed a polygon, ill-defined tumor, which had expansive growth with invasion of the spinal canal (Fig. 3A). The tumor removed at the subcutaneous level showed a polymorphic and unevenly distributed pattern with heavily pigmented spindle cells and fewer cells with eosinophilic nuclei. The extramedullary tumor consisted of a large nucleus, and there was no mitotic phase in the nucleolus either. Both lesions were surrounded by abundant cytoplasm containing coarse melanin granules. Immunohistochemical analysis revealed that the cells were strongly stained with melan-A (Fig. 3B) and human melanoma marker (HMB45) (Fig. 3C). The index ratio of Ki-67 positive cells was <2% (Fig. 3D). The diagnosis was invasive blue navei.

## 3. Discussion

Blue nevus are thought to be derived from melanocytes of neural crest cells that are arrested to the surface epithelium during embryonic migration.^[5]^Because of the same origination,blue nevus should be differentiated from menigeal melanoma and melanocytoma.^[4]^ Blue nevus has been divided into 2 histological variants, the common blue nevus and the cellular blue nevus.The more common type of blue nevus is a localized collection of variably pigmented, somewhat flattened or spindle cells with derdritic processes and arranged as irregular masses or bundles of cells separated by fanscicles of collegen. In contrast to the common blue nevus, cellular blue nevus consists of compact or oval cells with pale cytoplasm, which may contain little or no melamin. The cells are round and fusiform in shape. It tends to be larger and exhibits slower growth over time. Histologically, cellular blue nevus shows a biphasic pattern with lightly pigmented nodules comprising uniformly appearing spindle cells surrounded by sclerotic lesions containing more elongated, dendritic and heavily pigmented cells. Immunohistochemical staining is a valuable tool for the diagnosis of melanocytic neoplasms.

Strong expressions of S-100 and HMB45 were observed in the present case. Normally, cellular blue nevus rarely occurs in the central nervous system but is found most often on the extremities, scalp, and buttocks and exhibits a characteristic blue–black-gray–black clinical discoloration of variable sizes.^[1,2]^

Menigeal melanoma and blue nevus can be manifested as a large amount of pigment deposition, HMB45 and cell proliferation. However blue nevus have small heterogeneity and a lower Ki-67 index.

The case present in our study represent is special. The heterogeneity of the cells is not obvious, and there is no obvious mitotic phase or necrosis. Although the HMB45 is positive, the Ki67 index is low. The 2 lesions(subcutaneous and subdural) showed the same source during surgery. In summary, the diagnosis is invasive cellular blue nevus.

There have been 8 case reports in the literature on spinal cellular blue nevus so far(Table 1). In these reports, the cellular blue nevus was black with diffuse margins and has been described as both unifocal and multifocal.^[4–8]^ Although blue nevus is a benign tumor, it is generally reported that cellular blue nevus may have a low malignancy potential and can infiltrate lymph nodes in a pattern simulating melanoma.^[9]^ Therefore, the differential diagnosis of spinal blue nevus and malignant melanoma is key, and during the operation, we found that the tumor in the spinal canal is homologous with the tumor under the skin. The cellular heterogeneity of these 2 lesions was not obvious, and mitosis or necrosis was not observed. Although there was a large amount of pigment deposition with cell proliferation, it did not meet the histological diagnostic criteria of malignant melanoma. The cellular blue nevus can infiltrate deep tissue and even reach the spinal cord parenchyma, not really metastasis, but the likelihood of recurrence or malignancy may increase.^[4]^ Different from common pigmented nevus, cellular blue nevus can express HMB45, which is similar to malignant melanoma, but the ki67 index is very low, which is one of the key points for differentiation between cellular blue nevus and malignant melanoma.^[10]^

**Table 1:** Summary of reported cases of cellular blue nevus of the spine with published histopathology: Clinical features.

Author	Sex	Age	Growth focality	Location	Color	Margins	Malignant	Follow-up (mo)
KuRoKawa	Woman	40	Multifocal	Thoracic	Black	Diffuse	No	48
GRAHAM	Woman	46	Unifocal	Cervical	Black	Diffuse	Yes	24
	Woman	60	Unifocal	Cervical	Purple	Diffuse	Yes	Death
	Woman	52	Unifocal	Cervical	Grey	Clear	No	Unknown
Loghavi	Woman	55	Unifocal	Unknown	Unknown	Clear	No	8.4
B.Lach	Woman	20	Unifocal	Cervical	Black	Diffuse	No	36
Agarwalla	Man	54	Unifocal	Cervical	Black	Diffuse	No	60

## 4. Conclusion

The findings in our case show that surgical treatment is effective for treating cervical spinal cellular blue nevus without the need for postoperative radiotherapy and chemotherapy. Our case can illustrate that the absence of blue nevus on the skin surface cannot be used as an exclusion criterion for cellular blue nevus. In our case the relationship between 2 tumors and the clinical patterns of slow progression can be the basis of differential diagnosis from malignant melanoma, which helps to prevent excessive treatment and provides reassurance for the patient.

## Acknowledgments

We thank the patient and his family for participating in this case report and declare that there are no conflicts of interest. No financial support was received for the research, authorship, or publication of this manuscript.

## Author contributions

**Conceptualization:** Zhengwen Kang, QiuSheng Zhang.

**Data curation:** Zhengwen Kang, Bo Wang.

**Investigation:** Tong Wang.

**Methodology:** Shuwen Yang, Zetao Wu.

**Project administration:** QiuSheng Zhang.

**Visualization:** Mengyao Ma.

**Writing – original draft:** Xianfeng Rao, Jianwei Chen.

**Writing – review & editing:** Xianfeng Rao.

## References

[1] Rodriguez HA Ackerman LV. Cellular blue nevus Clinicopathologic study of forty-five cases. Cancer. 1968;21:393–405. 5637949 10.1002/1097-0142(196803)21:3<393::aid-cncr2820210309>3.0.co;2-k

[2] Zembowicz A Phadke PA. Blue nevi and variants: an update. Arch Pathol Lab Med. 2011;135:327–36. 21366456 10.5858/2009-0733-RA.1

[3] Barnhill RL Argenyi Z Berwick M. Atypical cellular blue nevi (cellular blue nevi with atypical features): lack of consensus for diagnosis and distinction from cellular blue nevi and malignant melanoma (“malignant blue nevus”). Am J Surg Pathol. 2008;32:36–44. 18162768 10.1097/PAS.0b013e3181573aaf

[4] Graham DI Paterson A McQueen A. Melanotic tumours (Blue Naevi) of spinal nerve roots. J Pathol. 1976;118:83–9. 943491 10.1002/path.1711180204

[5] Agarwalla PK Koch MJ Mordes DA. Pigmented lesions of the nervous system and the neural crest: lessons from embryology. Neurosurgery. 2016;78:142–55. 26355366 10.1227/NEU.0000000000001010

[6] Kurokawa R Kim P Kawamoto T. Intramedullary and retroperitoneal melanocytic tumor associated with congenital blue nevus and nevus flammeus: an uncommon combination of neurocutaneous melanosis and phacomatosis pigmentovascularis--case report. Neurol Med Chir (Tokyo). 2013;53:730–4. 24077274 10.2176/nmc.cr2012-0241 PMC4508742

[7] Loghavi S Curry JL Torres-Cabala CA. Melanoma arising in association with blue nevus: a clinical and pathologic study of 24 cases and comprehensive review of the literature. Mod Pathol. 2014;27:1468–78. 24743221 10.1038/modpathol.2014.62

[8] Lach B Russell N Benoit B. Cellular blue nevus (“melanocytoma”) of the spinal meninges: electron microscopic and immunohistochemical features. Neurosurgery. 1988;22:773–80. 3374792 10.1227/00006123-198804000-00030

[9] Magro CM Crowson AN Mihm MC. Unusual variants of malignant melanoma. Mod Pathol. 2006;19:S41–70. 16446716 10.1038/modpathol.3800516

[10] de la Fouchardiere A Blokx W van Kempen L C. ESP, EORTC, and EURACAN Expert Opinion: practical recommendations for the pathological diagnosis and clinical management of intermediate melanocytic tumors and rare related melanoma variants. Virchows Arch. 2021;479:3–11. 33432480 10.1007/s00428-020-03005-1

